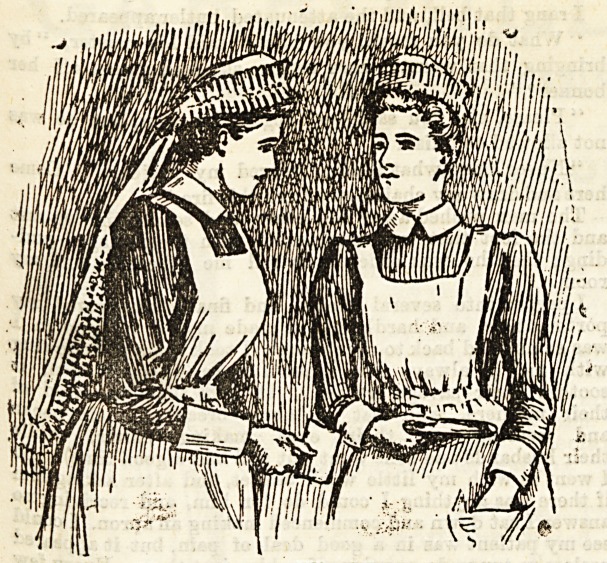# The Hospital Nursing Supplement

**Published:** 1892-04-09

**Authors:** 


*The Hospital*, April 9, 1892. Extra Supplement.

### "The Hospital" Nursing Mirror.

Being the Extra Nursing Supplement of "The Hospital" Newspaper.

Contributions for this Supplement should be addressed to the Editor, The Hospital, 140, Strand, London, W.C., and should have the word "Nursing" plainly written in left-hand top corner of the envelope.

### En Passant.

GOOD NEWS FROM SOUTH AFRICA.—Writing from Kimberley, S. Africa, to her friend and teacher, Mrs. Creighton Hale, a nurse says: "The hospital is in the best situation . . . . the rooms for the nurses are very good, and quite away from the hospital—they are under the charge of a lady, and there are two servants to make the beds, clean rooms, &c. Private nursing pays very well. . . . I have been to the Transvaal, Grahamstown, Port Elizabeth, Stort's Bay, and Cape Town, and have never had a day's illness in a year and three months. . . . I am the only midwife here so could not leave at present. If I stay three years I think I shall then go home, even if I come out again afterwards. There are five private nurses and they are always engaged."

SHORT ITEMS.—The Duchess of Albany hopes to attend the whole course of lectures to ladies on hygiene at the Parkes Museum.—Miss Kenealy is lecturing in Lincolnshire for the County Council under the Technical Education Act.—Two ladies have been nominated as guardians for Crewe.—Funds are required for the Chester Church Deaconesses' Institute, both for their Nursing Home and also for the Deaconesses' Home.—Miss Marsden has returned to St. Petersburg from Yakutsk, where she went to investigate the state of lepers, and to find out the truth of the rumoured cure in the shape of a herb medicine for leprosy.—The President of the Q. V. Jubilee Institute for Nurses wishes us to state that the salaries of nurses employed under that institute do not begin at *less* than £25 a year.

THE REGISTRATION OF NURSES.—The following letter from a member of the Association appeared in the *British Medical Journal* of 26th ult.:—

I see that, in addition to the new registration of nurses and midwives which the Royal British Nurses' Association is proposing to ask the Privy Council to charter—as set forth in the *British Medical Journal* of March 19th—Sir John Colomb has given notice of a resolution in Parliament that it is expedient to establish a general system of licence and registration for chimney-sweeps, with a view of preventing persons convicted of robbery or theft from practising the profession of chimney-sweeps.

I do not know what will be thought of this latest addition to public registers; but contrasting the wording of this resolution with that of the charter which you publish, it is evident that even chimney-sweeps will be far better protected than the nurses; at least, Sir John Colomb proposes to make a register which will have a Parliamentary sanction, and to prevent persons of evil life from assuming to be registered, and of protecting the public against such false assumption of title. In all these respects Sir John Colomb's register will be a real register; but that which it is proposed for the nurses is obviously a sham register—one which affords no protection to the public, since there is no penalty for falsely assuming whatever title such register may confer, nor any means for distinguishing between those who have the title and those who have not, and those who may have been put on it or struck off it.

It will be interesting to see how Sir Richard Quain or Sir Dyce Duckworth, to whom you appealed, will explain their apparent support of this parody of a register. But perhaps you are not aware that copies of a petition in favour of it have been circulated for signature on a printed form to the branches of the Association. Is not this rather an abuse?

We are asked to state that Lady Superintendents, Matrons, and other ladies engaged in the teaching and training of nurses throughout the country can obtain copies of the petition against registration for signature, on written application to the Treasurer, St. Thomas's Hospital, Albert Embankment, London, S.E.

THE NURSES' CO-OPERATION.—The General Committee meeting of the Nurses' Co-operation took place on Tuesday, and a variety of business was transacted. The association continues to grow, and the returns for each month in the present year testify to a steadily increased prosperity. To the constant applications from nurses wishing to join the Co-operation, the reply has still to be given that no more can be taken on until next autumn. The cordial good-feeling manifested by the Committee, and by those more intimately engaged in the affairs of the nurses, gives evidence that the welfare of the latter is the first object of the Co-operation.

NURSING HOMES AND PAYING PATIENTS.—The dinner in aid of the Hampstead Home Hospital and Nursing Institute brought in donations to the amount of £1,050. These paying homes do so much good work, and this Home, by its varied scale of charges, viz., from 7s. to five guineas a week, has a large scope. They do good by stepping in and giving those who are unable to procure good nursing in their own homes every aid to recovery for the payment of a certain sum; thus the patient avoids the public hospital, and many people are kept from going to a public hospital who can and ought to pay for their nursing. The Chairman at the Hampstead dinner paid a charming tribute to the nurses, and seemed relieved to feel that even "Sairey's" ghost at the sick man's side had practically become an impossibility.

RADCLIFFE INFIRMARY, OXFORD.—The Committee of this infirmary have made arrangements to receive a limited number of paying probationers at 30 guineas a year, who will receive board, lodging, laundress, and indoor uniform for this sum. They also receive probationers without payment who are required to give a year's service to the hospital after their probationary year has expired. Paying probationers desirous of serving an additional year have £10 of their original payment returned. Certificates will be given at the end of the first year to probationers who have proved their efficiency as nurses, and have passed their examinations satisfactorily, and a further and more complete certificate to those who have been there two years. We are glad to note that among the questions candidates for admission are required to answer is: "Are you strong and healthy, and have you always been so?" Satisfactory answers to this can never be insisted upon too rigorously. These new arrangements at the Radcliffe will, we hope, promote its progress in every way.

LINCOLN NURSES.—The fifth annual report of the Institution for Nurses at Lincoln is out and shows a good record of work during the past year. The Lincoln nurses, besides going to paying patients, go out at reduced rates to those who require skilled nursing, but who cannot afford to pay the full charge; twenty-four nurses have been supplied to these cases for sixty weeks, and in two cases no charge whatever was made. The institution also supplies district nurses for the poor in Lincoln, and they hope soon to have an additional nurse at work, and they have been training nurses in district work for the rural branch of Q.V.J. Institute. Funds for the two last mentioned branches of the work are badly needed, and so the Lincolnshire ladies are coming to the rescue with a bazaar in November, and if a lengthy list of patronesses goes any way towards success, money ought to pour in. It is always pleasant to hear one has not laboured in vain, so district nurses will be glad to hear the remark of a Lincoln clergyman: "One may visit a patient in the morning, leaving the home miserable and squalid, and on returning in the afternoon see an enormous difference, all brought about by a district nurse."

### On the Nursing of Children.

VIII.—OBSERVATION OF COLOUR, POSTURE, &c.

The obligation that exists for a nurse to observe accurately, and to report literally, the result of her observations, was touched upon in our first paper on "The Nursing of Children," but it is a subject which needs dwelling upon.

In the first place we must reflect that not all the details which we, as nurses, notice, need be formally reported to the doctor. In this as in many other cases, when to speak and when to hold silence is one of the puzzles which life sets before us.

With regard to colour, for instance, a nurse should learn much from this; text-books say that such and such a tint on a child's face means such and such a disease, and the comparatively raw probationer who has injudiciously wandered into one of these volumes, feels herself quite competent to pronounce on the complaint from which the new patient is suffering.

The wiser, because more experienced, nurse smiles at this whilst she awaits the doctor's coming, and when his diagnosis corresponds with her own suspicions, she feels an honest pride which, if she be discreet, she will keep to herself. Should her ideas prove faulty, she will set to work to think out what she can have omitted to observe which the doctor's practised eye so quickly discovered, and she will lay the lesson to heart for future guidance.

Now no nurse need tell a medical man what a child's colour is, but if it has definitely changed in the interval between his visits, then she can briefly state the hour when she first noticed the variation.

But when we turn to the subject of the cries of children, we come to a matter in which the nurse may be able, materially, to help the doctor, for it is not unusual for the little patient to lie still and silent whilst the physician is beside the bed, and therefore a description of the cries uttered during the rest of the twenty-four hours is absolutely essential. Thus it is well for nurses to train their ears to note all the varieties of sounds uttered by sick children, and a most interesting study they will find it, for not only has the kind of cry to be noticed, but also the frequency of it, whether it is uttered consciously, or if it also occurs during sleep, and many other points.

Then as regards posture, we have another wide field of interest, and the habit of observing the way in which children habitually lie soon becomes second nature to trained eyes. Occasionally we are fairly surprised by the rapidity and accuracy with which an experienced nurse can obtain information by a glance at a sleeping child.

Posture often helps greatly in the discovery of the onset of disease, and any little trick exhibited repeatedly by a boy or girl should be anxiously watched. Many a delicate spine would be strengthened by medical treatment if the first signs of weakness were understood by the child's friends. When the disease has become a certainty, they can probably describe graphically the attitude which was constantly assumed by the child, perhaps months before! But they say they "never thought that it meant anything"; and now that the weakness has become disease, and disease has developed deformity, the poor little victim is for the first time brought under the surgeon's eye. Alas! and alas! "preventable diseases" are more varied and numerous than we care to remember, and the innocent suffer for the carelessness, ignorance, and indolence of those to whose mercies their tender little bodies are entrusted.

In the out-patient department of any general hospital we soon grow to dread the sight of the poor little cases of hip disease, whose friends with rough clumsiness add so much to their pain. They are not wilfully unkind, but the oft-spoken remark, "He allers cries when I touches him," needs no comment, nor do the replies which the surgeon's questions elicit; the history of the first pain observed, the cries by night, and even the hasty words, and, perhaps, blows, administered because the poor mi te awakened other sleepers in the crowded and unsanitary d welling, the callous confession that this has gone on for months without medical treatment being sought for any alleviations of the little martyr's sufferings. All this shows an ignorance so lamentable that we are silenced by the greatness of the evil.

It may seem, at first, as if none of this trouble could be foreseen or prevented by nurses; but much lies in the power of those who do private or district work—for it is certain that every child with whom they come in contact is consciously or unconsciously criticised by trained eyes. Hence a word of warning, temperately and gently offered, is frequently gratefully received, and bears valuable and unexpected fruit.

In fact, it is oftener the case that too much weight is attached to a nurse's utterances instead of too little, and it therefore behoves her to speak with reserve and caution. One point which should always be reported by a nurse is the steady loss of flesh by a child. When she sees it is growing thin, she should always call attention to the matter—the doctor will know what amount of importance to attach to the fact, although he may not be the first to notice it, if the little patient's face remains unchanged.

It is sometimes said of the school girl or boy, "Oh! he is growing so fast, of course he is thin;" and this is probably a true explanation of the "lean and hungry" look, but it is for the mother or nurse at this period to bestow extra thought and care on the diet, which should be of a specially nourishing character, on the clothes, which should be warm and sufficiently light not to cause unnecessary fatigue, and on the surroundings of the child to see that they are of a perfectly hygienic nature both by night and day.

It needs tact and judgment to ensure sufficient supervision of girls and boys in their teens, whilst avoiding such anxious scrutiny of the health as may tend to foster the delicacy which we wish to escape from.

Sufficient employment, and diversity of interests, are the best safeguards against that self-contemplation and introspection to which young folks are prone; but we must beware of over-pressure, that evil outcome of modern education, over which so many arguments constantly take place, until some of us become doubtful, when the stream of words ceases, as to whether there exists a real, solid evil, or if it is not, sometimes, at any rate, a mere harmless broomstick which our nervous fancies have dressed up into the similitude of a very terrible bogey!

### Appointments.

Eton College Fever Sanatorium.—Miss Gertrude Moberley, who was trained as Lady Pupil at Guy's Hospital and has since been working at the New Hospital for Women, has been appointed Matron of the Eton College Fever Sanatorium.

Royal Naval Hospital, Stonehouse.—Sister Grace Mackay has been appointed Superintendent of this Hospital. She was trained at Bartholomew's, and was one of the first naval nursing sisters at Haslar ten years ago.

### The L.O.S. Examination.

The next class at the Midwives' Institute and Trained Nurses' Club for preparation for the London Obstetrical Society's examination will commence the last week in April. All enquiries as to dates, terms, etc, should be made to the Secretary, 12, Buckingham Street, Strand. The lectures at this institute will be resumed in May.

### Significant!

The *British Medical Journal* in its issue of the 19th ult. asked the Royal British Nursing Association the following pertinent questions regarding their proposed scheme for the registration of nurses :—

We notice that Sir Richard Quain and Sir Dyce Duckworth are among the proposed members of the Registering Council. We would ask them as authorities on registration to inform the profession on the points we have raised, and we put to them a few categorical questions, feeling sure that the profession will be glad of their answer.

1. What is to be the title of the registered nurses, and how is that title to be protected, and how also the public? What is the punishment of anyone who falsely assumes the title or continues to use it after being struck off the register? By what machinery are they to be tried before being struck off?

2. What means will the Registering Council possess of obtaining official evidence of the sufficiency of the curriculum training, and examinations of the existing or future training and teaching schools whose certificates are to be registered, and by what representative machinery can any such bodies be dealt with in case of failure of duty?

3. Has the assent of the leading training institutions in the metropolis to the creation of an official register and to the charter, been obtained? If not, how are its conditions to be applied to them? How is a representation of them to be assured under the charter?

4. What legal privileges will the registered nurses have over non-registered nurses?

The only answer forthcoming is the following letter which appeared in the *Journal* on the 2nd inst. :—

Sir,—In a letter signed "M.B.,M.A.," and also in an editorial note, both of which appeared in the *British Medical Journal* of March 26th, it was stated that copies of a petition in support of the Royal British Nurses' Association "have been forwarded to the branches of the British Medical Association for signature." As many of the best-known members of the "British Medical" are also members and strong supporters of the Nurses' Association, they may believe this statement, and naturally feel surprised that their particular Branches have not received the petition referred to. I must, therefore, request you to allow me to state that the assertion in question is entirely untrue.

I would take this opportunity to add that the Royal British Nurses' Association has no intention of carrying on a paper warfare, because its application for a Royal Charter is before the Privy Council, and is therefore *sub judice*. It has retained several eminent counsel, who will, if necessary, bring its case fully before that tribunal and the public, and prove how largely the medical profession support it, and how, why, and by whom it has been so strenuously opposed. There is, consequently, no useful object to be gained by a premature discussion in the press.—I am, &c.,

Bedford Fenwick,
Treasurer Royal British Nurses' Association.
Upper Wimpole Street, W.

The following note is appended to the above letter by the editor of the *British Medical Journal* :—

"We understand this to mean that the petition will not be further circulated for signature in the manner indicated; but we are informed that a printed copy of such petition was brought forward for signature recently at a branch meeting, and it was stated that such forms were to be forwarded elsewhere. We are aware—indeed our statement indicated—that medical support had been obtained for this application for the sanction of the Privy Council to a so-called "Register of Nurses," including midwives*, but our view is that so inadequate a document, and one so imperfectly safeguarded as to its titles and so devoid of authoritative means for legal protection of the public from false assumption of them, is unworthy of medical support, and likely to be more dangerous than useful to the public."

* The R.B.N.A. have abandoned the registration of Midwives, which finds no place in the charter submitted to the Privy Council.—Ed. T. H.

### THE HELPING HAND.

It is a great satisfaction when we are overworked or have a task of unusual difficulty, to find a helping hand to give us assistance, and perhaps pull us out of our troubles altogether. If we had fallen into a river and could not swim, how grateful we should be to the strong hand which landed us in safety on the shore, and the brave hand which at personal risk dragged us from before a nearing train, would never be forgotten. It comes more home to our present needs, however, to think what comfort a capable hand gives us in sickness. It dresses our wounds, renews our poultices and plaisters, remakes our beds and smoothes our tumbled pillows, and when we are growing stronger and begin to help ourselves, but are sitting half-dressed on the side of our couch, exhausted with the slight exertion, the welcome kind hand comes to the rescue and clothes us, and we feel once more in our right mind.

For help in these trials we may safely count on our fellow creatures, for it is seldom that we do not find assistance and consideration from them. But there are cases, especially in great bodily or mental anguish, when human love fails to solace us, and the truth of the words of a modern writer is borne in on our hearts, "Nothing but the Infinite pity is sufficient for the infinite pathos of human life." Then, only the Hand of God can aid us. But the pity and love which drew His Son down from Heaven to save the souls and succour and heal the bodies of His poor lost creatures, is as soft and warm and loving now as it was some eighteen hundred years ago, when

His strong arm triumphed o'er disease and death,
O'er darkness and the grave.

His touch in the old days brought life and health to all who sought it, and gave strength to the palsied limb, sight to the blind, and speech to the dumb. Shall we not then clasp the only Hand which can minister to the sinner's wounded soul and bring relief? The hands of Jesus, wounded for our sakes, are ever stretched out to draw us near to Him; we will clasp them, hold them fast, and be safe whate'er betides, for sin and Satan are powerless when we are grasped by Him, and "it is not easy to ruin one with whom the pressure of Christ's hand yet lingers in the palm." Our agony will be less keen when we are held by Him who bore like pains in His tender body for us, and who is helping us to bear them ourselves, and the misery of our hearts and the torments of an awakened conscience will be calmed and soothed by the strength of His own right hand, which alone gives us victory in our spiritual conflicts.

### Notes from Australia.

(*By Our Own Correspondent.*)

Melbourne, Jan. 29th.

There seems no probability of the Austin Hospital for Incurables fading into the background for lack of public attention, and there seems to be an ever fresh supply of complaints and mistakes to exercise the minds of the subscribers to that institution. The complaints at the weekly meeting ranged from allowing a moribund patient to leave the hospital, with death the consequence next day, down to the charge made by a female patient that her supply of gin was not regular! The report of the House Committee, recommending that visitors to the hospital, with the exception of clergymen, should be only admitted by the front door, was negatived very foolishly, considering that the hospital has four different entrances, which prevents the person in charge from ever being able to tell who is in the institution. Evidence was shown that persons got in who brought drink and other deleterious things to the patients, and, in fact, not so very long ago, several patients were made intoxicated, forty empty bottles being found to show the reason why. This is evidence which does not reflect credit on the observance of the nurses, and which sounds almost incredible. Pastor Herlitz proposed that as the public seemed to have lost all confidence in the Committee they should resign; this was seconded by the Rev. J. H. O'Connell, who was sure that the outside public thought it would be possible to get a better Committee; and, really, this sounds a moderate probability. The Chairman said he was certainly going to resign, as newspaper judgments and decisions on his conduct were things he was not going to allow; some of us over here are sensitive folk. A letter from a patient named Gay, recommending more care with regard to the treatment of the sputum of consumptive patients was read, and was answered by Mr. Harlin to the effect than an incinerator to destroy the sputum was to be obtained; and the complaint of the dirtiness of quilts and blankets was not substantiated by further evidence, but the facts that the consumptive patients are given no active treatment, and that such things as malt and cod liver oil are only administered when the patients ask for them, found nobody to answer them negatively.

The Chairman commented on the death of the consumptive patient, Thomas Wallis, who was allowed to leave the hospital with only a Chinese patient, in an almost dying condition and with no preparation made for his reception at his journey's end. The Matron, it appears, remonstrated with the man, saying he was not in fit condition to go; it would have been better to have sent for the doctor to put a stop to such a proceeding, for the old man after going some little way was met by the hospital Secretary, who took the unwilling patient back to the hospital, where he died next day. It was a question whether the Committee would have supported the Matron in retaining a patient against his will, as there was the case of Canon Cummins before the courts about a similar matter.

There seems to be a cruelly literal meaning given to the word "incurable" at this unhappy institution. A man can die anywhere: a hospital for incurables is a place where the dying may rest, and pass away as happily and painlessly as possible, if they are (as so few can realise themselves to be) really incurable. It sounds as though there were very little kindliness surrounding patients in a place where a nurse will tell a suffering man, "We have done all we can for you. You don't come here to be cured." This is not wonderful, perhaps, in an institution where the Matron states "that trained nurses are not necessary for incurables." Some of them appear, however, to be trained in hardness of heart pretty satisfactorily. It is to be hoped that the subscribers will take the matter into their own hands, and elect a committee who know what law and order mean.

It is a grain of comfort to hear that at the Board of Public Health meeting last week the number of cases of typhoid fever was declared to be less than for the corresponding two weeks of last year, but one hardly dares rejoice for fear that the measures for the prevention of the spread of the disease be relaxed in the least degree. The City Council have, however, made arrangements for the daily destruction of typhoid excreta by incineration, which is a practical step in the right direction.

Miss E. M. M'Kay and Miss Kate Chamberlain have been granted their applications for registration of 84, Curtain Street, Carlton, and 479, Latrobe Street, Melbourne, respectively, as private hospitals.

There has been a considerable stir at the inquest of a poor woman who died in the Women's Hospital last week from childbirth. She had been staying at the house of a midwife named Goldstein, but Dr. Fenwick, who was sent for to see her, treated her for her immediate wants, and left, returning in the afternoon with Dr. Newman Brown. Both endeavoured to confine the woman, but Dr. Fenwick had to leave, and Dr. Brown called in Dr. Parry, who in his turn called in Dr. Meyer, who recommended the removal of the woman to the Women's Hospital, where she died next day. The jury had to decide whether deceased died from causes over which Dr. Fenwick had no control, or whether he omitted to do certain things he should have done, or had done anything he ought not. Dr. Meyer, who finally delivered the woman at the hospital, declared that it was a case which absolutely needed manual or instrumental assistance. The decision was not arrived at in consequence of the absence of the midwife and Dr. Fenwick, and so the Coroner adjourned till February 2nd.

The report at the annual meeting of the Benevolent Asylum testified to the general depression which had had its effect on the funds of institutions. I am afraid this does not apply to Australia alone. The Committee gratefully acknowledged the assistance that the Charity Organization Society had afforded them both in preventing imposition and in getting those who had relatives in the asylum to give something to their support. The public subscriptions were commented on as small, but otherwise things seem to flourish here. The *Argus*, our chief daily paper, had a very strong article the other day on the Morgue returns of infanticide, which I mentioned in my last letter, or rather on the utter failure of the police to detect the criminals. It says that an agitation for the release of one convicted of this crime some two years ago shows how far impressionable people will go, and the police most likely think that it is excess of zeal to attempt to find the murderer. The article points out the necessity for establishing a foundling hospital, an institution which, far from encouraging crime, tends to decrease it. The Melbourne Hospital have decided to pass over the application of Dr. Beaney's relatives for part of the funds left them.

You have been complaining of the cold in England. Man is never satisfied. Here on the 22nd, the sun has been pouring down its blessings at the rate of 150 deg. outside.

The case of Canon Cummins, which I mentioned, was brought by Mr. Hayes, who applied for a writ of habeas corpus against Miss Morley, the Matron of the Austin Hospital, calling on her to show cause why she detained Canon Cummins in the hospital against his will. It appears that his son and his half-brother put him in, but paid for him out of a sum which his old parishioners allowed him, while the daughter knowing her father wished to leave wanted him to come out and go into a home of his own. However, the Matron refused flatly to lot Canon Cummins go without the consent of his son and half-brother, and I am glad to say the application was granted and the writ made returnable on the 28th inst. The hospital altogether is having an eventful time.

### Nursing Uniforms.

### IV.—LONDON HOSPITAL.

Many of our readers know by sight the pretty dark-green cloaks and bonnets that the private nurses of the London Hospital wear. This hospital has no outdoor uniform for the hospital nurses, but many of the sisters and nurses wear a cloak and a bonnet and some add a veil.

Sisters' indoor uniform consists of navy blue cachemir dress, an apron with a high bib crossing over at the back, and caps trimmed with a lace-edge frill and long "tails" at the back.

The Staff Nurses have striped cotton dresses, a linen apron like the Sisters, and small caps of jaconette muslin trimmed with Coventry frilling.

Probationers wear checked cotton dresses, aprons with square cut bibs, and caps similar to the Staff Nurses, but the edging is different.

### News from Jamaica.

In the current number of "Service for the King," is an interesting letter from a Deaconess at Jamaica. She writes: "At the invitation of the Bishop of Jamaica, two deaconesses were sent from the Deaconess House, Mildmay Park, one to take charge of the training of parochial workers, the other the training of nurses. They landed in Kingston on August 8th, 1890, where a nice large airy house had already been obtained in one of the best streets for a centre home.

The idea of trained women doing public work for God is still new in Jamaica, therefore candidates for training have come forward slowly. They are not received into the home from one class in society only, neither need they be of one shade of opinion, but all must have personal piety of a decided character.

Ladies who desire to be Deaconesses, receive eighteen months' parochial training, and six months' in the Public Government Hospital, where Sister K. has two wards under her charge.

Those ladies and superior young women who desire to be nurses are receiving a thorough training. We have also some respectable coloured women, whom we call parochial helpers, who, having been sent by their clergy from different parishes in the island, receive six months' training in nursing, and nine months' parochial teaching.

The need of skilled nurses is greatly felt in Jamaica; the few women who were trained in the now defunct Lady Barkley Institution have almost died out; and in the country parts cases often prove fatal through want of efficient nurses to carry out the doctor's orders.

At the present time there are three ladies and two young coloured women in training in the hospital; one of the latter has already been employed twice in nursing private cases."

### Everybody's Opinion.

*[Correspondence on all subjects is invited, but we cannot in any way be responsible for the opinions expressed by our correspondents. No communications can be entertained if the name and address of the correspondent is not given, or unless one side of the paper only be written on.]*

### HOLIDAY EXPERIENCES.

"A Nurse" writes: After the experience of many years in holiday taking under various circumstances, I have come to the conclusion that a really successful holiday is much more doubtful of achievement than it should be, for nurses at any rate; such an occasion is a real epoch through which we reckon to gain health, pleasure, and new interests. The latter are, I think, especially necessary for nurses whose minds get sadly limited to the sphere of the sick room or institution, to the restricting of their mental vigour and power of useful development. In all occupations it would seem that variety from the usual routine constitutes the principal refreshment. There are, as a rule, the limitations of money, time, and experience in the case of nurses, and it struck me, now that Easter, the opening of the holiday season is approaching, that some readers of The Hospital might afford great assistance, if they would offer suggestions for holiday trips, with a few necessary details, where all the three limitations I have mentioned are allowed for. We, many of us, have, of course, private friends, who are only too glad to welcome and make much of us, and a nurse, at the end of a long period of work, invariable in the case of an institution nurse, before the holiday comes round, is very often not inclined for enterprise on her own behalf. The first invitation we receive, therefore, appears to us to offer the desired haven, and despite the kindest intentions of our friends, we, many of us, return with a vague sense at best, that we might have had a better time elsewhere. It is an effort for which we are not prepared, to conform, when on relaxation bent, to the clearly-defined rules of some households. We have had enough of living by rule, of conforming to the ways of others, and we want to do what we like, and go where we like, as fancy suggests at the moment. Then, again, homes which many of us, thank God, possess, are not calculated always to meet all the requirements of the case for the whole of our holiday. The locality is not healthy in one case, or the circumstances of the family are not such as to afford us a cheering change in another. In such cases it is unwise not to supplement our visit home by some other form of change. Our best friends will be alive to the importance of a health-giving holiday, and will not take our action as a proof of want of affection, but aid us in satisfying our particular needs. The less fortunate of us sympathise with the complaint we often hear—"I want a change sadly, but I don't know where to go." Still not a few have had happy experiences from time to time, and if they would give us the benefit of their good fortune by telling us how to follow their example, they would earn the thanks of many.

### Notes and Queries.

### Queries.

A "Contributor" would feel extremely obliged if any reader could tell her if there are any "homes" where a lady, about 50, who wants rest, change, and country or seaside change, could be taken free. She has no means, could attend herself, and give a little assistance to invalids or others in many ways (she has been overworked, and has heart affection), either permanently or for the summer. Any information about a home or hospital called the "Prendergast Home," and any information on the subject will be gladly received.

### Answers.

*Enquirer.*—Playfair's "Midwifery" is the standard book, but it is rather dear, £1 8s.; try and get a second-hand copy at Kimpton's, 82, High Holborn. Barnes' "Manual of Midwifery" is very good too.

*Nurse L.*—"First Lines in Midwifery," by Dr. G. E. Herman, is published by Cassell and Co.; do not know the price.

*Jidica.*—The Hertford British Hospital, Rue de Villers, Levallots Perret, Seine, is the only one we know of for certain as to probationers; they advertised for one in our columns last week. The Levick Home, Madame Neilson's Institution, and the St. George's Association take trained nurses. See "Hospital Annual" for further information.

*G. Michell.*—Your communication received. Shall have attention next week.

### A Troublesome Case.

"I wish you would oblige me by taking this case," said my Lady-Superintendent as she came into the nurses' sitting-room with an open note in her hand, "I am afraid the patient is exceedingly tiresome, for his last nurse has left him because he spoke roughly to her, and they want one who will be 'firm and kind, and not put out by his fits of temper.'" Here my artful Superintendent looked at me in an appreciative way, as much as to say "I think you are about the only nurse on the staff who is equal to this case." Now she knew perfectly well that I was a "woman's woman," and that I particularly objected to nursing members of the opposite sex. It was not that they gave more trouble or anything of that sort, but men as a whole are a selfish and uncertain lot, well or sick, and the craven way that they give up hope and think they're sure to die when they are moderately ill, and the fuss they make about a finger-ache, irritates me to such a degree that it upsets my constitution. Why, a woman has a hundred times more pluck!

However, I did not altogether dislike the idea of taking the case. I would show this wretched man that one nurse in the world was not afraid of him. I would be kind—yes, very kind—and firm, and not put out; but at the same time I should contrive by my superior attitude to point out to him the error of his ways, so I packed my portmanteau with that air of quiet determination that invariably precedes success. As I came downstairs I heard the voice of the nurse, who did not like being spoken roughly to, saying, "He is an awful patient; she will be back again to-night." Little did she know the spirit that animated *me*.

On reaching my destination the door was opened by an abnormally thin butler, who directed a small footman to carry in my luggage. In the hall I was met by a worried-looking lady, who said that she "hoped I should find everything comfortable," but she evidently did not expect it. As she was speaking the electric bell sounded, and the small footman flew upstairs four steps at the time, leaving the butler gazing at my portmanteau as if it were a coffin and he wondered why he wasn't inside.

Then a voice like a tempest came rushing downstairs and nearly blew my bonnet off. It was something between a bellow and a roar, and the words were, apparently, "Send that woman up here."

I was almost carried upstairs by the united efforts of the butler and footman. At the the top of the broad flight of steps one of the men opened the door of the invalid's room, while the other cautiously pushed me inside. On the opposite side of the room, just in front of a large fire, sat my patient, his tall form reposing in an invalid's chair, and his leg supported on a rest, while tied over the toes of the said leg was something that resembled a loosely-made dumpling in a white cloth.

"Sit down," he said gruffly, and I sat down. He put on his pince-nez, and looked at me sarcastically. I looked at him also, but kindly. I was not put out, in fact, I was comfortably thinking that here was a curious specimen of that degenerate genus man, and that I should dissect him, analyse him, turn him inside out, and generally enjoy finding out all his hypertrophied bad qualities.

"What are you thinking about," he shouted at me at last.

"I am just considering how long it will be before you are roasted through," I said kindly.

"Oh, the dickens, you are!" he said. "Ring that bell." I rang that bell, and the attenuated butler appeared.

"What do you mean, you idiot," said his master, "by bringing that woman here before she had taken off her bonnet?"

"Please, sir, you said——" began the man, but he was not allowed to finish.

"Never mind what I said," roared my patient. "Come here and draw my chair away from this fire."

The man pushed away the chair, and settled the leg-rest and the foot that was ornamented with the damaged pudding; then he was ordered to tell me where to find my room.

I peeped into several rooms, and finally discovered my portmanteau, and hardly had I made myself tidy when I was summoned back to my patient. I took some plain sewing with me (I always sew when I have to nurse men, it soothes their savage breasts. It puts them in mind of their mothers, who sat at home breaking their backs and wearing out their eyes making shirts, while their husbands and sons went out and had a good time). So I went in with my little work basket, and after asking him if there was anything I could do for him, and receiving no answer I sat down and commenced making an apron. I could see my patient was in a good deal of pain, but it appeared useless to try to do anything for him just then. Every few minutes he seemed to get a bad twinge in his gouty foot, and then he would groan and rumple up the few white hairs left on his head.

Presently he asked me for the *Times* newspaper, but unfortunately I did not catch the word "Times," and seeing several daily papers on the table I asked him which he required. A bad twinge seemed to take him just at the moment, and almost purple with rage and pain, he seized a small cushion that lay by his side and flung it violently, not at me, but at the opposite wall, where I had previously noticed a large bare space. My face was fixed to look as if I thought it quite usual for elderly gentlemen to throw cushions at walls, but I had not bargained with my sense of the ridiculous. All of a sudden the funniness of the whole affair came over me, and sitting down in the nearest chair I laughed till my sides ached, and the tears ran down my cheeks, and really, if I had suppressed my laughter I am sure I should have injured myself internally. Presently I heard my patient growling and understood that he was asking "What the Dickens I was up to now," and I felt obliged to tell him between my gasps, that he really looked so funny with his hair on end—and the cushion—and that awful poultice on his poor toe—that I could not help myself, but I was dreadfully sorry and would not do it again. We were very quiet for a long time after that; I went on with my sewing, and he lay back in his chair with his eyes shut, but the expression of his face was not unkindly. Finding he was still uneasy, I ventured presently to leave the room and get the materials to make a poultice. Very softly I went back and very gently I removed the pudding and replaced it with my nicely-made poultice. Then I put the leg-rest at the right angle and got him to let me arrange his pillows comfortably, and then, because I was afraid I had hurt his feelings by laughing, I took one of his hands in mine and stroked it softly and he looked quite pleased.

I did not go back to the home that night, in fact, I stayed away a great many weeks and never was nurse so petted and pampered, as I was by my gruff old patient and everybody in the house. I had found out that it drove the master wild to know that people were afraid of him, and I managed to keep him in good humour by alternately laughing at him and humouring him; as his gout improved so did his temper, and the butler and the footman are no longer attenuated, but have become quite fat and happy specimens of humanity.

### Where to Go.

The Whitechapel Picture Exhibition is now open in St. Jude's Schools, and is well worth a visit. It is really intended for East Londoners, and the Hon. Secretary hopes that those who go and like it will tell others that it is open.

These few summer days make Londoners begin to think of how to spend half-holidays in the country, and we hope, every now and then to be useful in suggesting good places to go to within easy access.

## Figures and Tables

**Figure f1:**
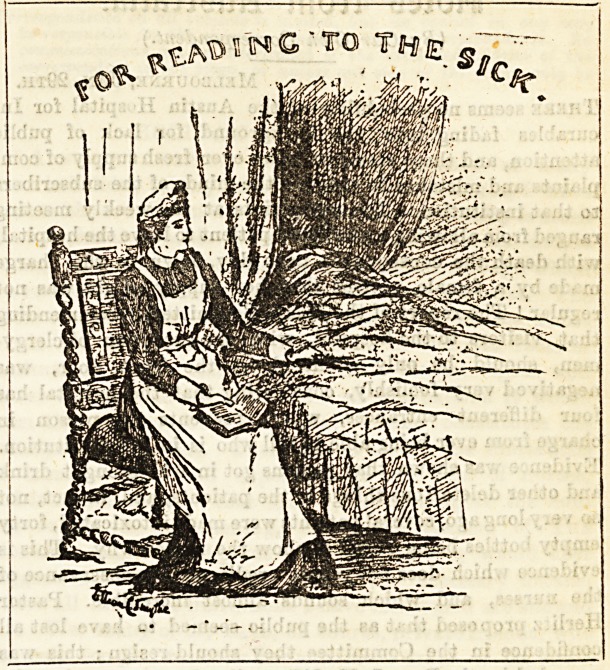


**Figure f2:**